# Intraoperative remimazolam-nalbuphine infusion reduces visceral traction response during cesarean delivery: a randomised trial

**DOI:** 10.1186/s12871-026-03909-6

**Published:** 2026-05-18

**Authors:** Juan Meng, Xi-Shu Deng, Run Han, Shan Jiang, Yu-Chen Zou, Tao Zou, Jian-Gui Gong, Hong-Mei Bao, Si-Ting Wang, Suwas Bhandari, Bo Feng, Li-Ming Cheng

**Affiliations:** 1 Department of Anesthesiology, Kunming City Maternal and Child Health Hospital, Kunming, Yunnan 650031 China; 2 Department of Paediatric Intensive Care Unit, Kunming Children’s Hospital, Kunming, Yunnan 650100 China; 3 Department of Anesthesiology, Kunming Children’s Hospital, Kunming, Yunnan 650100 China; 4 Department of Anesthesiology, Kunming First People’s Hospital, Kunming, Yunnan 650224 China; 5 Department of Anesthesiology, The Second People’s Hospital of Yunnan Province, Kunming, Yunnan 650021 China; 6 School of International Studies, Wenzhou Medical University, Wenzhou, Zhejiang 325027 China

**Keywords:** Remimazolam, Nalbuphine, Visceral traction response, Cesarean delivery, Spinal anesthesia

## Abstract

**Background:**

Visceral traction response (VTR) occurs in over 40% of cesarean delivery under spinal anesthesia. This study evaluated the efficacy and safety of intraoperative remimazolam combined with nalbuphine for attenuating VTR.

**Methods:**

In this prospective, randomised, double-blind, placebo-controlled trial, 72 parturients undergoing elective cesarean delivery under spinal anesthesia received either remimazolam (0.05 mg/kg) with nalbuphine (0.1 mg/kg) (R + N group, *n* = 36) or normal saline (Placebo group, *n* = 36) after umbilical cord clamping. The primary outcome was positive traction response incidence.

**Results:**

The R + N group demonstrated significantly lower positive traction response incidence (5.6% vs. 41.7%, *p* < 0.001) and reduced VTR scores during abdominal exploration and suturing. The incidence of nausea (22.2% vs 58.3%, *p* = 0.002) and chest tightness (8.3% vs 36.1%,* p* = 0.005) was significantly lower in the R + N group. Maternal satisfaction was higher (9.56 ± 0.88 vs. 7.11 ± 1.47, *p* < 0.001). No significant hemodynamic differences or serious adverse events occurred.

**Conclusions:**

Remimazolam combined with nalbuphine significantly reduces VTR during cesarean delivery, providing effective sedation and analgesia while maintaining hemodynamic stability, representing a safe adjunct to neuraxial anesthesia.

**Trial registration:**

Chinese Clinical Trial Registry ChiCTR2500108913, registered 9 September 2025.

**Supplementary Information:**

The online version contains supplementary material available at 10.1186/s12871-026-03909-6.

## Background

Cesarean delivery is one of the most frequently performed surgical procedures worldwide [1]. However, visceral traction response (VTR) remains a common and distressing complication during cesarean delivery under spinal anesthesia, with a reported incidence exceeding 40% [2]. This response is primarily triggered by peritoneal traction, manipulation of intra-abdominal organs, and uterine contractions, manifesting clinically as visceral pain, nausea, vomiting, and abdominal discomfort [3, 4], beyond compromising maternal comfort and interfering with surgical procedures. VTR may induce hemodynamic instability, cardiac arrhythmias, and increased oxygen consumption, potentially leading to adverse maternal and fetal outcomes in severe cases [5]. Furthermore, inadequately managed intraoperative discomfort has been associated with long-term psychological sequelae, including post-traumatic stress disorder and postpartum depression [6].

Several pharmacological strategies have been employed to prevent VTR during cesarean delivery. Intrathecal or epidural opioids have demonstrated efficacy in attenuating this response; however, their use is frequently complicated by respiratory depression, urinary retention, pruritus, and delayed gastric emptying, limiting their widespread application [7]. Esketamine, while effective in preventing VTR, is associated with undesirable cardiovascular effects, including tachycardia and hypertension, as well as psychomimetic adverse events such as dizziness and hallucinations [2]. These limitations underscore the need for alternative pharmacological approaches that provide adequate visceral analgesia and sedation while maintaining hemodynamic stability and minimising adverse effects.

Nalbuphine, a mixed κ-opioid receptor agonist and partial μ-opioid receptor antagonist, offers a promising alternative for visceral pain management [8–10]. It provides moderate analgesic efficacy with particular effectiveness against visceral pain, exhibits a ceiling effect for respiratory depression, maintains hemodynamic stability, and carries lower addiction potential compared to pure μ-opioid agonists [11]. Remimazolam is a novel ultrashort-acting benzodiazepine characterised by rapid onset (1–2 min), short duration of action, and predictable recovery (10–40 min) [12–14]. Unlike traditional benzodiazepines, remimazolam undergoes rapid hydrolysis by tissue esterases independent of hepatic and renal function, producing inactive metabolites with minimal risk of accumulation. Recent studies have demonstrated that remimazolam effectively reduces anxiety [15], provides adequate sedation, and decreases the incidence of postoperative nausea and vomiting in parturients undergoing cesarean delivery [16].

We hypothesised that the combination of nalbuphine and remimazolam would synergistically attenuate VTR by targeting both the analgesic and anxiolytic-sedative components of this multifactorial response. The κ-opioid receptor agonism of nalbuphine may specifically address visceral nociception, while the sedative and anxiolytic properties of remimazolam may modulate the emotional and autonomic components of VTR. This study aimed to evaluate the efficacy and safety of intraoperative remimazolam combined with nalbuphine in reducing VTR during cesarean delivery under spinal anesthesia. Secondary objectives included assessment of sedation levels, hemodynamic stability, adverse events, and maternal satisfaction.

## Methods

### Study design and ethical approval

This prospective, single-centre, randomised, double-blind, placebo-controlled trial received ethical approval from the Ethics Committee of Kunming Maternal and Child Health Care Hospital (Approval No. Kun Fu You Lun Shen [2025] No. 2). The trial was carried out between September 9 and December 9, 2025, and was registered with the Chinese Clinical Trial Registry (ChiCTR2500108913). The study adhered to the Declaration of Helsinki [17] and the Consolidated Standards of Reporting Trials (CONSORT) guidelines [18]. Written informed consent was obtained from all participants prior to their inclusion in the study.

### Participants

Patients scheduled for elective cesarean delivery at our institution were recruited for this study. Inclusion criteria were: (1) American Society of Anaesthesiologists (ASA) classification II-III; (2) age 18–40 years; (3) gestational age ≥ 36 weeks; and (4) planned cesarean delivery under spinal anesthesia.

Exclusion criteria were: (1) contraindications to spinal anesthesia; (2) severe hepatic or renal dysfunction; (3) cardiopulmonary diseases; (4) severe obstetric complications including uterine rupture, cord prolapse, preeclampsia, or eclampsia; (5) body mass index ≥ 35 kg/m^2^; (6) failed spinal anesthesia requiring conversion to general anesthesia; (7) anticipated blood loss > 1500 mL; and (8) known allergy to study medications.

### Randomisation and blinding

Participants were randomly allocated in a 1:1 ratio using a computer-generated sequence with a block size of 4. Allocation concealment was ensured via sequentially numbered, opaque, sealed envelopes prepared by an independent researcher who had no role in patient care or data analysis. To maintain blinding, an independent anesthesia nurse, uninvolved in the study, prepared the study drugs in identical, opaque syringes labeled with coded numbers. Solution A contained either nalbuphine (1 mg/mL) or an equivalent volume of normal saline, and Solution B contained either remimazolam (1 mg/mL) or an equivalent volume of normal saline. The anesthesiologist administering the drugs and the surgical team remained blinded to group allocation. To minimize the potential for functional unblinding, sedation depth was monitored using standardized criteria, and the anesthesiologist responsible for drug administration and patient monitoring was not involved in data collection. All subjective outcomes were assessed by an independent investigator who was blinded to group allocation and absent during the induction and maintenance phases. Unblinding occurred only after the completion of data collection and statistical analysis.

### Anesthesia management

Upon arrival in the operating room, standard monitoring was established, including pulse oximetry, non-invasive blood pressure, and continuous electrocardiography. Intravenous access was secured, and 500 mL of Ringer's lactate solution was administered as a preload. Supplemental oxygen was provided via face mask at 5 L/min throughout the procedure. With the patient in the left lateral decubitus position, a lumbar puncture was performed at the L3-L4 interspace using a 25-gauge pencil-point spinal needle. Upon confirmation of cerebrospinal fluid flow, 1.7–2.2 mL of 0.75% isobaric ropivacaine was administered intrathecally. The patient was then positioned supine with a 15° left lateral tilt. Sensory block level was assessed using pinprick testing, and surgery commenced once a T4-T6 dermatomal level was confirmed. All patients received patient-controlled intravenous analgesia (PCIA) postoperatively. The analgesic solution consisted of sufentanil 100 μg diluted in 100 mL of normal saline. PCIA settings included a background infusion rate of 2 mL/h, a bolus dose of 2 mL, and a lockout interval of 15 min.

Adverse events were continuously monitored and managed according to predefined protocols. Hypotension, defined as mean arterial pressure (MAP) < 65 mmHg or > 20% decrease from baseline, was treated with phenylephrine 50–100 μg intravenously. Hypertension, defined as MAP > 100 mmHg or a > 20% increase from baseline, was managed with intravenous urapidil 5–10 mg. Bradycardia, defined as a heart rate < 60 beats/min, was treated with atropine 0.3–0.5 mg intravenously. Tachycardia, defined as heart rate > 100 beats/min, was managed conservatively with observation or esmolol if symptomatic. Nausea and vomiting were treated with ondansetron 4 mg intravenously. Hypoxemia, defined as SpO₂ < 90%, was managed with airway manoeuvres and assisted ventilation if necessary.

### Interventions

The study medications were administered at two designated time points. Immediately after fetal delivery, Solution A was administered intravenously at 0.1 mL/kg, corresponding to nalbuphine 0.1 mg/kg in the R + N group or an equivalent volume of normal saline in the placebo group. Following maternal-infant bonding, Solution B was administered as an initial bolus of 0.05 mL/kg followed by continuous infusion at 0.1 mL/kg/h until surgical completion, corresponding to remimazolam 0.05 mg/kg bolus plus 0.1 mg/kg/h infusion in the R + N group or equivalent volume of normal saline in the placebo group.

### Outcome measures

The primary outcome was visceral traction response during cesarean delivery, assessed using a 4-point scale: Grade 0, no discomfort; Grade 1, mild discomfort without visceral pain, nausea, or vomiting; Grade 2, mild visceral pain and nausea; Grade 3, severe visceral pain with nausea, vomiting, and abdominal distension. Positive traction response was defined as Grade ≥ 2 at any assessment time point.

Secondary outcomes included Ramsay Sedation Scale score, hemodynamic parameters (MAP and heart rate) measured at three time points (T1: fetal delivery; T2: abdominal exploration; T3: muscle layer closure), incidence of adverse events (nausea, vomiting, chest tightness, gastric spasm, hypotension, bradycardia, tachycardia, and hypoxemia), and patient satisfaction score assessed using a 0–10 numerical rating scale.

### Sample size calculation

The sample size was calculated based on the primary outcome, the incidence of VTR during cesarean delivery. Previous studies have reported that the incidence of VTR under spinal anesthesia is approximately 48.6% [2]. We hypothesized that the remimazolam-nalbuphine (R + N) intervention would achieve a 50% relative reduction in the incidence of VTR, a magnitude of effect consistent with the efficacy of other potent anesthetic adjuncts reported in recent literature [2, 3]. Using a two-sided α of 0.05, a statistical power of 80%, and accounting for a 10% potential dropout rate, PASS 15.0 software (NCSS, LLC, Kaysville, UT, USA) determined that a total of 72 participants (36 per group) were required.

### Statistical analysis

Statistical analyses were performed using SPSS version 25.0 (IBM Corp., Armonk, NY, USA). The Shapiro–Wilk test was used to assess the normality of continuous variables. Normally distributed continuous data were expressed as mean ± standard deviation and compared using an independent samples t-test. Non-normally distributed continuous data were presented as median with interquartile range and compared using the Mann–Whitney U test. Categorical variables were expressed as frequencies and percentages and analysed using the chi-square test or Fisher's exact test when the expected cell frequency was less than 5.

To account for the longitudinal nature of the data collected at multiple time points, we employed a repeated-measures approach. For hemodynamic parameters (MAP and HR), which were normally distributed, repeated-measures ANOVA was used to evaluate the interaction between group and time. For non-normally distributed ordinal data (Traction Response Score and Ramsay Sedation Score), Generalized Estimating Equations (GEE) were utilized to assess the interaction effect. All statistical tests were two-sided, and *p* < 0.05 was considered statistically significant.

## Results

### Patient enrolment and baseline characteristics

**Fig. 1:**
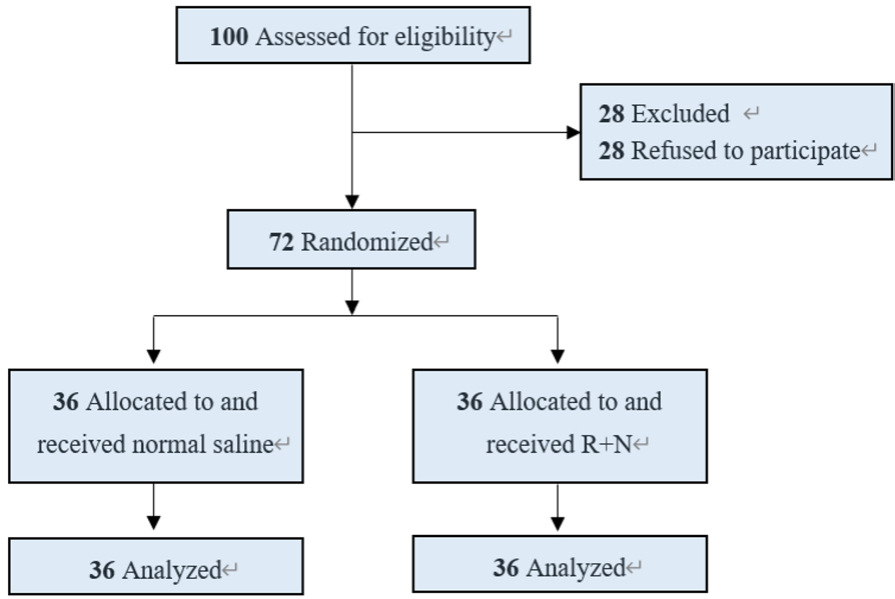
Participant flowchart

A total of 100 patients undergoing elective cesarean delivery were assessed for eligibility. Twenty-eight patients were excluded because they declined to participate, leaving 72 patients who were randomised into two groups: the Remimazolam–Nalbuphine (R + N) group (*n* = 36) and the Placebo group (*n* = 36). All randomised patients completed the study, and no patients were lost to follow-up. The CONSORT flow diagram is presented in Fig. 1. Baseline demographic and clinical characteristics were comparable between the two groups (Table 1). There were no statistically significant differences in age (28.47 ± 4.52 vs. 30.17 ± 4.18 years, *p* = 0.097), height (158.50 ± 5.16 vs. 158.58 ± 4.67 cm, *p* = 0.942), weight (68.05 ± 6.72 vs. 70.04 ± 6.68 kg, *p* = 0.206), or BMI (27.12 ± 2.56 vs. 27.87 ± 2.49 kg/m^2^, *p* = 0.205). The distribution of ASA classification was similar between groups (*p* = 1.000), with the majority of patients classified as ASA II. Gestational age (38.6 ± 1.0 vs. 38.8 ± 0.8 weeks, *p* = 0.345), intraoperative fluid volume (1073.61 ± 56.68 vs. 1080.56 ± 58.89 mL, *p* = 0.610), and estimated blood loss (296.11 ± 87.78 vs. 318.06 ± 85.91 mL, *p* = 0.281) were also comparable between the two groups.

**Table 1:** Baseline Characteristics of Patients

Variables	R + N Group (*n* = 36)	Placebo Group (*n* = 36)	*p* value
Age (years)	28.47 ± 4.52	30.17 ± 4.18	0.097
Height (cm)	158.50 ± 5.16	158.58 ± 4.67	0.942
Weight (kg)	68.05 ± 6.72	70.04 ± 6.68	0.206
BMI (kg/m^2^)	27.12 ± 2.56	27.87 ± 2.49	0.205
ASA Classification [n (%)]			1.000
Class Ⅱ	35 (97.2%)	36 (100%)	
Class Ⅲ	1 (2.8%)	0 (0%)	
Gestational age (weeks)	38.6 ± 1.0	38.8 ± 0.8	0.345
Intraoperative fluid volume (mL)	1073.61 ± 56.68	1080.56 ± 58.89	0.610
Blood loss (mL)	296.11 ± 87.78	318.06 ± 85.91	0.281

### Primary outcome: visceral traction response

The visceral traction response scores are presented in Table 2. At baseline (T1, fetal delivery), the traction response scores were similar between groups, with median scores of 0 (0, 0) in both groups (*p* = 0.414). However, significant differences emerged at subsequent time points. At T2 (abdominal exploration), the R + N group demonstrated significantly lower traction response scores compared to the Placebo group [median 0 (0, 0) vs. 1.5 (1, 2), *p* < 0.001]. This difference persisted at T3 (muscle layer suturing), with the R + N group maintaining lower scores [median 0 (0, 0) vs. 1 (1, 2), *p* < 0.001]. The incidence of a positive traction response (defined as a score ≥ 2 at any time point) was significantly lower in the R + N group than in the Placebo group (5.6% vs. 41.7%, *p* < 0.001), representing an absolute risk reduction of 36.1%.

**Table 2:** Comparison of primary and secondary outcomes

Outcomes	Time Point	R + N Group (n = 36)	Placebo Group (n = 36)	*p* *Between*	*p* *(Interaction)*
Traction Response Score	T1	0 (0, 0)	0 (0, 0)	0.414	** < 0.001**
	T2	0 (0, 0)	1.5 (1, 2)	** < 0.001**	
	T3	0 (0, 0)	1 (1, 2)	** < 0.001**	
Positive Traction Response [n (%)]	-	2 (5.6%)	15 (41.7%)	** < 0.001**	
Ramsay Sedation Score	T1	2 (2, 2)	2 (2, 2)	0.821	** < 0.001**
	T2	4 (4, 4)	2 (2, 2)	** < 0.001**	
	T3	4 (4, 4)	2 (2, 2)	** < 0.001**	
Patient Satisfaction Score	-	9.56 ± 0.88	7.11 ± 1.47	** < 0.001**	
MAP (mmHg)	T1	75.44 ± 14.38	76.83 ± 11.21	0.452	0.614
	T2	74.19 ± 11.22	73.44 ± 10.20	0.648	
	T3	71.53 ± 12.19	71.72 ± 10.55	0.912	
HR (beats/min)	T1	81.28 ± 9.72	85.28 ± 10.29	0.085	0.482
	T2	85.14 ± 8.86	84.03 ± 12.08	0.655	
	T3	82.75 ± 10.73	86.47 ± 13.39	0.192	

### Secondary outcomes

Ramsay Sedation Scores were comparable between groups at T1 [median 2 (2, 2) in both groups, *p* = 0.821]. Following the administration of study drugs, the R + N group achieved significantly higher sedation levels at both T2 [median 4 (4, 4) vs. 2 (2, 2), *p* < 0.001] and T3 [median 4 (4, 4) vs. 2 (2, 2), *p* < 0.001], indicating adequate sedation without excessive drowsiness.

Hemodynamic stability was maintained throughout the procedure in both groups. There were no significant differences in mean arterial pressure (MAP) between the R + N and Placebo groups at T1 (75.44 ± 14.38 vs. 76.83 ± 11.21 mmHg, *p* = 0.452), T2 (74.19 ± 11.22 vs. 73.44 ± 10.20 mmHg, *p* = 0.648), or T3 (71.53 ± 12.19 vs. 71.72 ± 10.55 mmHg, *p* = 0.912). Similarly, heart rate (HR) remained comparable between groups at all time points: T1 (81.28 ± 9.72 vs. 85.28 ± 10.29 beats/min, *p* = 0.085), T2 (85.14 ± 8.86 vs. 84.03 ± 12.08 beats/min, *p* = 0.655), and T3 (82.75 ± 10.73 vs. 86.47 ± 13.39 beats/min, *p* = 0.192).

Patient satisfaction scores were significantly higher in the R + N group compared to the Placebo group (9.56 ± 0.88 vs. 7.11 ± 1.47, *p* < 0.001).

### Adverse events

The incidence of adverse events is summarised in Table 3**.** The total incidence of adverse events was significantly lower in the R + N group compared to the Placebo group (33.3% vs. 77.8%, *p* < 0.001). Notably, the R + N group demonstrated a significantly lower incidence of nausea (22.2% vs. 58.3%, *p* = 0.002), chest tightness (8.3% vs. 36.1%, *p* = 0.005), and gastric spasm (0% vs. 19.4%, *p* = 0.011). Although numerically lower in the R + N group, the incidences of hypotension (8.3% vs. 13.9%, *p* = 0.710), tachycardia (11.1% vs. 22.2%, *p* = 0.206), and vomiting (8.3% vs. 13.9%, *p* = 0.710) did not reach statistical significance. Bradycardia occurred in only one patient in the Placebo group (2.8%, *p* = 1.000). No episodes of hypoxemia were observed in either group.

**Table 3:** Comparison of adverse events

Adverse Events	R + N Group (*n* = 36)	Placebo Group (*n* = 36)	*p* value
Hypotension	3 (8.3%)	5 (13.9%)	0.710
Tachycardia	4 (11.1%)	8 (22.2%)	0.206
Bradycardia	0 (0%)	1 (2.8%)	1.000
Hypoxemia	0 (0%)	0 (0%)	-
Nausea	8 (22.2%)	21 (58.3%)	**0.002**
Vomiting	3 (8.3%)	5 (13.9%)	0.710
Chest tightness	3 (8.3%)	13 (36.1%)	**0.005**
Gastric spasm	0 (0%)	7 (19.4%)	**0.011**
Total incidence	12 (33.3%)	28 (77.8%)	** < 0.001**

## Discussion

This randomised, double-blind, placebo-controlled trial demonstrated that intraoperative remimazolam, combined with nalbuphine, significantly attenuated the visceral traction response during cesarean delivery under neuraxial anesthesia, reducing the incidence from 41.7% to 5.6%, while improving patient satisfaction and decreasing adverse events.

Visceral traction response remains a significant challenge during cesarean delivery under regional anesthesia, with reported incidences of more than 40% [2]. The pathophysiology involves vagal afferent fibres and splanchnic nerves transmitting nociceptive signals via pathways incompletely blocked by conventional neuraxial anesthesia [19, 20]. Additionally, prostaglandins, serotonin, and inflammatory mediators released during surgical manipulation activate visceral afferent pathways [21, 22]. The 41.7% incidence in our placebo group aligns with previous literature [2].

Various pharmacological strategies have been investigated, yet each presents notable limitations. Dexmedetomidine causes bradycardia and hypotension [3]; ketamine produces psychotomimetic effects [23]; propofol lacks intrinsic analgesia and potentiates hypotension [24]; intrathecal opioids carry respiratory depression risks [25]. Therefore, safer alternatives are urgently needed. The R + N combination offers complementary pharmacological mechanisms. Remimazolam, a novel benzodiazepine with a context-sensitive half-time of 7–8 min, undergoes rapid esterase-mediated hydrolysis, making it advantageous for obstetric anesthesia [26]. Its reversibility with flumazenil provides additional safety margins [27]. Nalbuphine, a mixed κ-agonist/μ-antagonist opioid, offers adequate visceral analgesia through κ-receptors while exhibiting a ceiling effect on respiratory depression [9, 28]. This combination provides anxiolysis and central modulation via remimazolam, while nalbuphine targets visceral nociceptive pathways.

The Ramsay Sedation Scores of 4 achieved at T2 and T3 indicate optimal sedation, allowing maternal comfort during manipulation while preserving maternal-infant bonding capacity [29, 30]. In this study, the target sedation depth of Ramsay score 4 was chosen to optimise maternal comfort during visceral manipulation while preserving spontaneous respiration and airway reflexes. Notably, no episodes of hypoxemia or respiratory depression occurred, indicating that remimazolam combined with nalbuphine maintains adequate respiratory function even at this sedation level. Furthermore, the timing of drug administration, after cord clamping, ensured that maternal-infant bonding, early breastfeeding contact, and neonatal safety were not affected. Critically, hemodynamic stability was maintained throughout the procedure [31], with comparable MAP and HR between groups, unlike propofol or dexmedetomidine, which may potentiate hypotension.

In our study, the overall incidence of adverse events in the placebo group was 77.8%. While this figure may appear high, it reflects the significant baseline burden of visceral traction-related symptoms-such as nausea, chest tightness, and gastric discomfort-that frequently occur during cesarean delivery under spinal anesthesia in the absence of prophylactic adjuncts. Previous studies have consistently documented that surgical manipulation of the uterus and peritoneum can trigger vagal-mediated responses in 48.6% to 79.7% of patients [2, 3, 32, 33]. Our findings are consistent with this clinical reality, where the placebo group experienced a high frequency of transient, self-limiting symptoms directly attributable to unmitigated surgical stimulation. Consequently, the high incidence observed in our control group underscores the clinical necessity for effective intraoperative management strategies to mitigate these common, yet distressing, adverse events.

The R + N group exhibited a significantly lower incidence of intraoperative nausea compared to the control group (22.2% vs. 58.3%). Despite similar hemodynamic profiles, this difference is likely due to the effective attenuation of the VTR. During cesarean section, VTR is a primary trigger for nausea via vagal stimulation; the synergistic effect of remimazolam and nalbuphine appears to blunt this response. Furthermore, nalbuphine’s unique pharmacological profile—acting as a kappa-opioid receptor agonist and a mu-opioid receptor antagonist—is associated with a lower incidence of opioid-induced nausea and vomiting compared to pure mu-agonists [34, 35]. This is supported by clinical evidence in parturients, where nalbuphine demonstrated superior anti-emetic properties compared to other opioids or placebo [34, 36]. Collectively, the sedative and analgesic synergy of the study drugs likely mitigated the surgical stress response, contributing to the reduced incidence of nausea. Furthermore, the reduced incidence of nausea in the intervention group may be partially attributed to the sedative and analgesic effects of the remimazolam-nalbuphine infusion. Sedation can elevate the threshold for symptom perception and mitigate anxiety-related exacerbation of nausea during visceral traction. While this mechanism may influence the subjective reporting of symptoms, it represents a significant clinical benefit that enhances the overall intraoperative experience for the parturient, thereby addressing the high baseline burden of visceral discomfort observed in our control group.

The sedative and analgesic synergy of remimazolam and nalbuphine attenuated the overall surgical stress response, which is a recognized contributor to intraoperative nausea. The fact that hemodynamic parameters remained similar between groups suggests that the reduction in nausea was not secondary to hypotension-induced cerebral ischemia, but rather a direct result of the blunted visceral afferent signaling and the optimized analgesic-sedative state. The significantly higher satisfaction scores in the R + N group (9.56 ± 0.88 vs. 7.11 ± 1.47) reflect the comprehensive benefits of reduced discomfort and fewer adverse events. Enhanced perioperative experience may contribute to positive birth experiences and potentially reduce postpartum psychological sequelae [37, 38].

The clinical implications of these findings are significant. By substantially reducing the visceral traction response and enhancing sedation comfort, the remimazolam-nalbuphine regimen improves the maternal intraoperative experience and minimises procedure-related stress. Diminished visceral discomfort also facilitates smoother surgical conditions, enabling obstetricians to operate with fewer interruptions and thereby enhancing overall operative workflow. Importantly, effective management of intraoperative distress may yield downstream psychological benefits, as maternal discomfort during cesarean delivery has been associated with an increased risk of postpartum anxiety, traumatic recall, and depressive symptoms. Consequently, this pharmacological strategy offers benefits that extend beyond the intraoperative period and may contribute to a more positive overall childbirth experience.

### Limitations

This study has several limitations that should be acknowledged. First, it was conducted at a single centre, which may limit the generalisability of the findings. Second, the absence of single-agent comparator arms (remimazolam alone or nalbuphine alone) precludes determining each drug's individual contribution to the observed effects. Third, although medications were administered after cord clamping, we did not perform long-term neonatal follow-up, nor did we collect systematic neonatal outcome data (e.g., Apgar scores, umbilical artery pH), which are essential for confirming the safety profile. Fourth, the fixed-dose regimen does not address whether titrated doses might achieve similar efficacy with fewer adverse effects, highlighting the need for future dose–response studies.

Finally, a potential limitation is the possibility of functional unblinding. Given the distinct sedative profiles of the intervention, the clinical team may have been aware of the group allocation, which could theoretically influence the assessment of subjective outcomes such as visceral traction response and nausea. However, to mitigate this bias, we utilized independent, blinded outcome assessors for all primary and secondary endpoints. Furthermore, as discussed in recent studies, while sedation depth may vary between groups, the use of standardized assessment tools remains the standard approach to minimize such bias. Therefore, our findings should be interpreted with caution, and future large-scale, multi-centre trials are warranted to confirm these results and comprehensively evaluate neonatal safety.

## Conclusions

Intraoperative administration of remimazolam-nalbuphine significantly reduces the incidence of visceral traction response during cesarean delivery, offering a favorable profile of sedation and analgesia with hemodynamic stability. While these findings suggest that this combination is a promising adjunct to neuraxial anesthesia, the results should be interpreted with caution due to the single-centre design and limited sample size. Further large-scale, multi-centre trials are warranted to confirm these findings and to comprehensively evaluate neonatal safety outcomes.

## Supplementary Information

Supplementary Material 1.

## Data Availability

The datasets used and/or analysed during the current study are available from the corresponding author upon reasonable request.

## Abbreviations

VTR: Visceral traction response
ASA: American Society of Anesthesiologists
PCIA: Patient-controlled intravenous analgesia
MAP: Mean arterial pressure
HR: Heart rate
